# Is Gene Drive Research Losing Traction?

**DOI:** 10.4269/ajtmh.25-0242

**Published:** 2025-08-12

**Authors:** Gregory C. Lanzaro, Ana M. Kormos

**Affiliations:** University of California Malaria Initiative, University of California Davis, Davis, California

## Abstract

Significant progress has been made in developing gene drives, especially for mosquito vectors of malaria. It is widely agreed that a critical next step in advancing this technology is to evaluate it through small-scale field trials. However, obtaining permission to move forward with these trials has stalled, threatening this potentially transformative line of research. In this paper, roadblocks delaying progress are identified from the perspective of a developer group tasked with translating this technology to the field. We suggest that groups engaged in long-running discussions about risk and the formulation of a global regulatory framework are hindering progress. This is because these groups conflate large-scale deployment with small-scale trials, which have very different risk landscapes. Here we argue that confined field trials are essential for accurately assessing risk and should be conducted soon, with regulation by authorities in the country in which they will be conducted.

In 2021, Ethan Bier published a review on the status of gene drive research titled “*Gene Drives Gaining Speed*.”^1^ In it, he reviewed the significant progress being made, primarily by groups engaged in gene drive research aimed at eliminating malaria from Africa. Progress in the laboratory led to the initiation of activities at sites across Africa, including sites in Tanzania (Transmission Zero), Burkina Faso, Ghana, Uganda (Target Malaria), and São Tomé and Príncipe (University of California Malaria Initiative). These activities occur at sites believed to be environmentally contained and include entomological and parasitological studies, capacity building, and robust engagement programs. These studies involve preparations for a field trial of gene drive mosquitoes as one of their principal goals. Despite this activity, permission to conduct initial field trials has not been obtained. Failure to move forward with this critical next step threatens this promising line of research. In this paper, the primary issues that we believe contribute to this roadblock are discussed: 1) a bewildering array of published guidelines, recommendations, and injunctions, 2) conflation of regulatory issues surrounding large-scale deployment with small-scale contained field trials, 3) reliance of local authorities on guidance from organizations that lack adequate knowledge or expertise, 4) confusion about which entities hold regulatory authority, and 5) local questions concerning financial incentives and long-term financial impacts on disease prevention programs.

Progress in the laboratory and field work has outpaced progress made around the conference table. The large social science and public health policy community that has emerged around gene drives is mired in seemingly endless discussions about risk and ethical issues. There is certainly no question that considering ethics and risk is essential for the responsible use of gene drive technology; however, interminable discussions about these issues need to be productive, and in our opinion, they currently are not. What has been accomplished is the publication of a host of overly long, complex, and redundant “soft-governance” guidelines.^2^^–^^13^ According to their authors, these documents were designed to assist developers and local regulatory authorities in moving the field forward; in this regard, they have failed. What they have achieved is promoting confusion and fear among institutional administrators, regulators, funders, and parts of the public. The authors of many of these documents state the need to engage local communities in decision-making while issuing written statements about how to do this without involving “local communities” themselves.^14^ In fact, these groups envision establishing an international organization to create rules to control gene drive research with statutory authority to enforce the rules.^5^^,^^15^ All of this is impeding progress toward field trials and resulting in the stagnation of this potentially transformative and lifesaving line of research.

Existing guidance and discussions on the regulation and risk assessment of gene drives are largely based on the broad international influence and transboundary, large-scale deployment of the technology across Africa. There is little discussion or consideration of the immense differences between this and small-scale, contained field trials. Such trials, as recommended by the WHO,^3^ are a critical next step in informing broader application and regulation. With little attention from the research community on what is minimally required for this advance, researchers remain mired in rounds of negotiations and debate. Meanwhile, local regulators and government leaders look to “technical experts” for guidance where there are none.

Our experience as developers seeking to conduct a confined, small-scale trial shows that the external expertise and guidance that national decision-makers seek are largely unavailable or nonexistent. Local WHO officers know little about their own agency’s published guidelines for testing genetic technology for malaria,^3^ and politicians and regulators are unfamiliar with or struggle to engage with other international agencies that have published guidance on gene drives. Large international forums and meetings are called to discuss risk and regulation for gene drives, but these are often out of reach geographically, linguistically, and economically for many who should be at the center of these conversations. Requests for consultation and advice go unanswered or are set aside because of limited knowledge, resulting in further delays and the cultivation of concern.

What is required for a confined gene drive field trial, and who will decide? Ultimately, the first gene drive field trial will likely be determined by leaders in a country that boldly chooses to be the first to apply the technology. It is these leaders who will hold the authority and shape how researchers approach the risk and governance of the technology. The gene drive community at large should support leaders willing to consider taking this bold step forward. Unfortunately, decision-makers are overwhelmed by guidance that lacks a clear and concise list of actionable items, checklists, or instructions for those who are not technical experts.

Malaria control programs in Africa rely on external funding to maintain and support personnel, infrastructure, and general operations. The 2024 WHO World Malaria Report states that $4 billion was spent on malaria control and elimination in 2023. Gene drive developers have been questioned about the long-term financial incentives and support that will follow if gene drive technology is applied and successfully leads to malaria elimination. How will this affect the millions of dollars allocated to existing malaria control programs? Will some of this money remain available for ongoing surveillance, infrastructure, and operations? Currently, there are no financial incentives offered to countries for implementing gene drive technology, nor do malaria funding sources appear to have a clear position on the use of a technology that could lower long-term costs, increase efficacy, and improve health outcomes. A clear position from malaria program funders on the use of the technology and the available funding to support malaria programs in the post-release surveillance and monitoring programs would be helpful.

A lack of progress risks inducing fatigue among donors, developers, and stakeholders. If gene drive research and development are to proceed, progress must be demonstrated and soon. Field trials have been identified as the logical next step.^3^ In addition to addressing issues related to genetically engineered [GE] mosquito efficacy (fitness, mating competitiveness, dispersal, etc.), field trials will advise risk assessments, inform regulators, contribute to refining GE products, and improve mathematical modeling. Careful selection of sites for field trials should mitigate many of the perceived risks associated with larger-scale gene drive deployment.^14^ Confined field sites include physical islands^16^^,^^17^ or ecological islands within the mainland, and these are abundant.^18^^–^^21^

The next significant step toward demonstrating progress in the application of gene drive for malaria elimination is to perform small-scale, ecologically contained field trials, which urgently need to proceed. There have been enough discussions and debates; it is time to move forward. To this end, the following recommendations are presented:

- Clear message of support from the WHO and malaria control program funding agencies for conducting a confined field trial to generate evidence necessary for advancing this technology;
- WHO guidance for a testing checklist for political leaders and decision-makers considering the application of this technology: what is minimally required to move forward from the WHO’s perspective;
- Replace meetings and discussions about international regulatory frameworks and guidance with statements of support for national authorities in developing regulations appropriate for a small-scale release in their country;
- Gene drive developers, funders, and related agencies encourage those with regulatory authority to move ahead with a regulatory process that leads to a timely “Go/No Go” decision for field trials.

Malaria remains a significant public health threat in sub-Saharan Africa, and it is clear that currently applied methods for its control are not adequate to achieve elimination. Genetically engineered mosquitoes with gene drive offer a potentially cost-effective, environmentally responsible, sustainable, and equitable approach for controlling malaria and, when used in conjunction with existing methods, eliminating it. We need more voices to speak out and emphasize that potential risks must be balanced with potential benefits, and must encourage those regulatory authorities with the power to approve field trials to do so. At the risk of doing nothing new, we risk losing another 600,000 lives to malaria in 2025.
